# Integrated multi-omics analysis provides insights into genome evolution and phosphorus deficiency adaptation in pigeonpea (*Cajanus cajan*)

**DOI:** 10.1093/hr/uhac107

**Published:** 2022-05-17

**Authors:** Chun Liu, Yuling Tai, Jiajia Luo, Yuanhang Wu, Xingkun Zhao, Rongshu Dong, Xipeng Ding, Shancen Zhao, Lijuan Luo, Pandao Liu, Guodao Liu

**Affiliations:** Tropical Crops Genetic Resources Institute, Chinese Academy of Tropical Agricultural Sciences, Haikou 571101, China; College of Forestry & College of Tropical Crops, Hainan University, Haikou 570228, China; BGI Institute of Applied Agriculture, BGI-Shenzhen, Shenzhen 518120, China; School of Life Science, Anhui Agricultural University, Hefei 230036, China; Tropical Crops Genetic Resources Institute, Chinese Academy of Tropical Agricultural Sciences, Haikou 571101, China; College of Forestry & College of Tropical Crops, Hainan University, Haikou 570228, China; College of Forestry & College of Tropical Crops, Hainan University, Haikou 570228, China; Tropical Crops Genetic Resources Institute, Chinese Academy of Tropical Agricultural Sciences, Haikou 571101, China; Tropical Crops Genetic Resources Institute, Chinese Academy of Tropical Agricultural Sciences, Haikou 571101, China; BGI Institute of Applied Agriculture, BGI-Shenzhen, Shenzhen 518120, China; College of Forestry & College of Tropical Crops, Hainan University, Haikou 570228, China; Tropical Crops Genetic Resources Institute, Chinese Academy of Tropical Agricultural Sciences, Haikou 571101, China; Tropical Crops Genetic Resources Institute, Chinese Academy of Tropical Agricultural Sciences, Haikou 571101, China

## Abstract

Pigeonpea (*Cajanus cajan*) is an important legume food crop and plays a crucial role in a secure food supply in many developing countries. Several previous studies have suggested that pigeonpea has great potential for phosphorus (P) deficiency tolerance, but little is known about the underlying mechanism. In this study, the physiological and molecular responses of pigeonpea roots to phosphate (Pi) starvation were investigated through integrating phenotypic, genomic, transcriptomic, metabolomic, and lipidomic analyses. The results showed that low-Pi treatment increased total root length, root surface area, and root acid phosphatase activity, and promoted the secretion of organic acids (e.g. citric acids, piscidic acids, and protocatechuic acids) and the degradation of phospholipids and other P-containing metabolites in the roots of pigeonpea. Consistent with the morphological, physiological, and biochemical changes, a large number of genes involved in these Pi-starvation responses were significantly upregulated in Pi-deficient pigeonpea roots. Among these Pi-starvation response genes upregulated by low-Pi treatment, four gene families were expanded through recent tandem duplication in the pigeonpea genome, namely *phosphate transporter 1* (*PHT1*), *phosphoethanolamine*/*phosphocholine phosphatase* (*PECP*), *fasciclin-like arabinogalactan protein* (*FLA*), and *glutamate decarboxylase* (*GAD*). These gene families may be associated with Pi uptake from the soil, phospholipid recycling, root morphological remodeling, and regulation of organic acid exudation. Taken together, our results suggest that pigeonpea employs complex Pi-starvation responses to strengthen P acquisition and utilization during low-Pi stress. This study provides new insights into the genome evolution and P deficiency adaptation mechanism of pigeonpea.

## Introduction

As a macronutrient, phosphorus (P) contributes to the growth and development of plants and is found in many key biomolecules, including nucleic acids, phospholipids, and energy sources (ATP and NADPH). Plants are able to directly absorb P from soils as soluble inorganic phosphate (Pi) [1]. However, Pi is easily immobilized by organic compounds, iron (Fe), or aluminum (Al) oxides/hydroxides present in soils into forms that cannot be absorbed by plants [2]. Pi deficiency is a major limiting factor for crop growth and productivity throughout ~70% of global cultivated land [3]. A faster growth in demand for Pi fertilizers in modern agriculture will result in increased production costs, soil degradation, and water eutrophication [4]. Hence, it is very important to perform basic research on physiological, biochemical, and molecular responses developed by plants during Pi limitation, as well as develop P-efficient crop cultivars.

In order to keep Pi homeostasis during Pi starvation, plants have evolved numerous adaptation mechanisms designed to enhance P acquisition efficiency (PAE) and P utilization efficiency (PUE) [5]. The strategies for strengthening PAE include altering root architecture and morphology, symbiosis with arbuscular mycorrhizal fungi, exuding organic acids from roots into the rhizosphere, release of acid phosphatase (APase) and ribonuclease from roots, as well as expressing Pi-starvation-inducible (PSI) genes, such as Pi transporters [6, 7]. Some major PUE-related strategies for plants include recycling Pi from internal P reservoirs, replacing membrane phospholipids with non-phosphorus lipids (e.g. galactolipids and sulfolipids), and facilitating a glycolytic bypass to minimize the requirement for ATP and ADP [8]. A complex signaling network is able to orchestrate diverse responses in plants against Pi deficiency [9].

High-throughput ‘omics’ technologies have been widely used to investigate plant responses to low-Pi stress. Transcriptome sequencing and analysis have been a powerful tool to identify Pi-starvation response (PSR) genes in plants, such as *Arabidopsis thaliana* [10], rice (*Oryza sativa*) [11], and soybean (*Glycine max*) [12]. Meanwhile, metabolome analysis reveals that Pi deficiency has broader impacts on a variety of metabolites, such as amino acids, organic acids, phospholipids, sugars, and phenylpropanoids [12, 13]. However, the plant’s response to Pi starvation is a complex process and network; therefore, an integrated multi-omics approach, including genomics, transcriptomics, proteomics, and metabolomics, can be a promising route to obtain a comprehensive picture of the PSR.

Pigeonpea (*Cajanus cajan*) is the sixth most important legume food crop globally, with ~5 000 000 ha of planting area [14]. Due to excellent productivity under extreme environmental conditions such as heat, drought, and low soil fertility, pigeonpea is widely grown in many developing countries in the tropical and subtropical regions using poor agricultural practices [15]. Importantly, pigeonpea is the main protein source, and therefore plays a vital role in the secure supply of food and nutrition in these developing countries [15]. It is well known that pigeonpea has increased ability to acquire and utilize P when subjected to Pi deficiency [16], yet the underlying molecular mechanisms of how the plant adapts to low-Pi stress remain largely unknown. The pigeonpea genome has been available for a decade [14], and provides a foundation to analyze low-Pi stress tolerance traits in pigeonpea along with its genomic evolution. Thus, the aim of this study was to investigate global changes in gene expression and metabolite accumulation in roots of pigeonpea responding to Pi starvation by integrating genome, transcriptome, and metabolome analyses. This will contribute to increasing our understanding of the mechanisms behind the adaptation of pigeonpea to Pi deficiency.

## Results

### Morphological and physiological changes in responses to Pi starvation

Pigeonpea seedlings were grown under low-Pi (−Pi, 0 mM KH_2_PO_4_) or high-Pi (+Pi, 0.3 mM KH_2_PO_4_) conditions to observe the effect of Pi deprivation on growth. The results showed that shoot fresh weight (FW), shoot dry weight (DW), shoot P content, and root P content were significantly decreased in −Pi treatment compared with +Pi treatment (Supplementary Data Fig. S1). However, −Pi treatment resulted in a significant increase in root FW, root DW, total root length, and root surface area by >24% (Supplementary Data Fig. S1). In addition, the root-internal APase activity and root-associated APase activity were 1.9- and 2.6-fold higher, respectively, in −Pi treatment than in +Pi treatment (Supplementary Data Fig. S1).

### Genome-wide analyses of tandem-duplicated genes and transcriptomic responses to Pi starvation in pigeonpea roots

**Figure 1:**
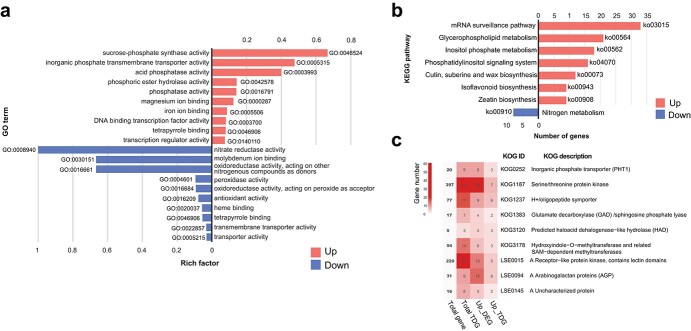
Transcriptome analysis of pigeonpea roots in response to Pi starvation. **a** GO enrichment analysis of up- and downregulated genes under low-Pi conditions. **b** KEGG pathway enrichment analysis of up- and downregulated genes under low-Pi conditions. **c** KOG enrichment analysis of TDGs (Total TDG), upregulated DEGs (Up_DEG), and upregulated TDGs (Up_TDG). KOG items that are significantly enriched in all three categories are shown.

Pigeonpea roots under −Pi and + Pi conditions were collected and sequenced to investigate the gene expression patterns in response to Pi deficiency. A total of 43 Gb (average 7.3 Gb per sample) of clean data with Q20 >97.0% were obtained after quality control (Supplementary Data Table S1). We mapped clean reads onto the pigeonpea genome [14], resulting in a genome mapping ratio >87%. As a result, there were 39 029 genes expressed in −Pi and/or + Pi conditions (Supplementary Data Table S2). Furthermore, a total of 1772 differentially expressed genes (DEGs) were identified in pigeonpea roots under low-Pi conditions, and a higher number of genes were upregulated (1241 upregulated and 531 downregulated; Supplementary Data Fig. S2). Among the 5334 novel protein-coding genes, there were 176 DEGs (Supplementary Data Table S3). Following Gene Ontology (GO) and Kyoto Encyclopedia of Genes and Genomes (KEGG) enrichment analysis, upregulated genes were significantly enriched in GO terms ‘sucrose-phosphate synthase activity’, ‘inorganic phosphate transmembrane transporter activity’, ‘APase activity’, ‘phosphoric ester hydrolase activity’, and ‘phosphatase activity’ (*P*_adj_ < 0.05), as well as being enriched in KEGG pathways of mRNA surveillance and glycerophospholipid metabolism (*P*_adj_ < 0.05) (Fig. 1a and b; Supplementary Data Tables S4 and S5). In addition, genome-wide identification of tandem-duplicated genes (TDGs) in pigeonpea was performed, and revealed 3382 TDGs in the pigeonpea genome, 231 of which belonged to DEGs (167 upregulated and 64 downregulated; Supplementary Data Table S6). Furthermore, eukaryotic orthologous groups (KOG) enrichment analysis of TDGs, upregulated DEGs, and upregulated TDGs showed that nine gene families belonged to all three categories simultaneously, including inorganic phosphate transporter, glutamate decarboxylase/sphingosine phosphate lyase, predicted haloacid dehalogenase-like hydrolase, arabinogalactan proteins, etc. (*P*_adj_ < 0.05) (Fig. 1c; Supplementary Data Tables S7–S9).

### Metabolomics and lipidomics analyses of pigeonpea root responses to Pi starvation

**Figure 2:**
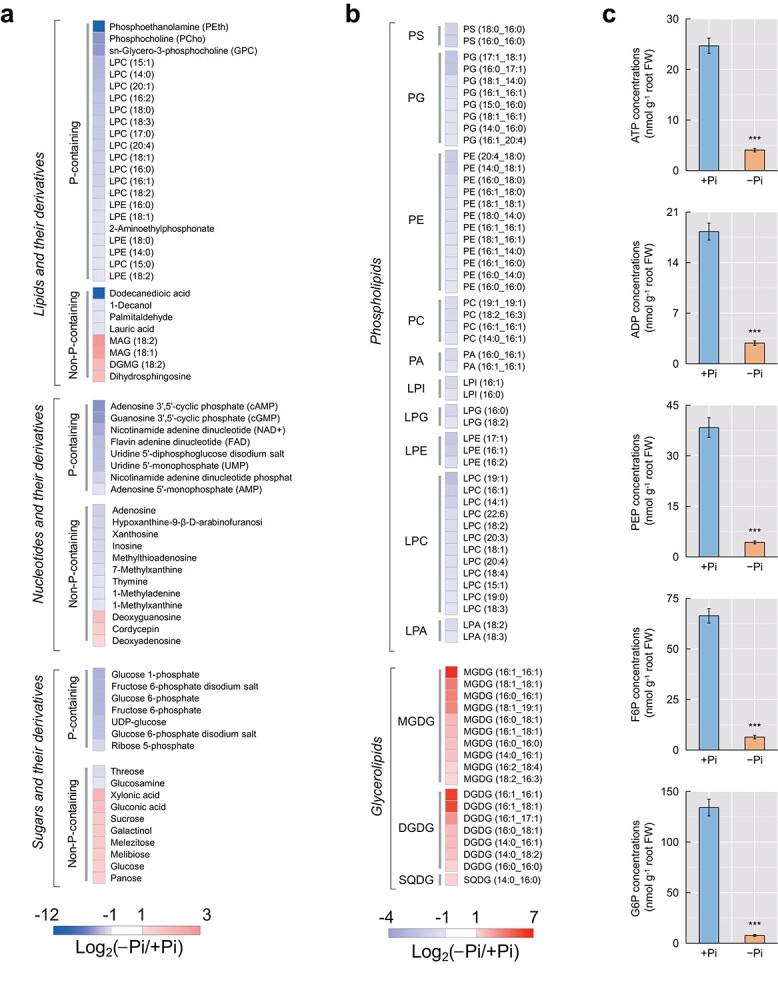
Changes in the accumulation of P-containing metabolites in the roots of pigeonpea seedlings grown under low-Pi versus high-Pi conditions. **a** DAMs containing phosphate groups identified by widely targeted metabolomics analysis. Variations in metabolites are represented by fold changes calculated using the formula log_2_(−Pi/+Pi). **b** Differentially accumulated phospholipids and glycerolipids identified by lipidomics analysis. Variations in lipids are represented by fold changes calculated using the formula log_2_(−Pi/+Pi). PS, phosphatidylserine; PG, phosphatidylglycerol; PE, phosphatidylethanolamine; PC, phosphatidylcholine; PA, phosphatidic acid; LPI, lysophosphatidylinositol; LPG, lysophosphatidylglycerol; LPE, lysophosphatidylethanolamine; LPC, lysophophatidylcholine; LPA, lysophosphatidic acid; MGDG, monogalactosyldiacylglycerol; DGDG, digalactosyldiacylglycerol; SQDG, sulfoquinovosyl diacylglycerol. **c** Targeted detection of five P-containing metabolites in the roots of pigeonpea. Data are means ± standard error of five biological replicates. Asterisks indicate significant differences between −Pi treatment and +Pi treatment according to Student’s *t*-test: ^***^P < .001. ATP, adenosine 5′-triphosphate; ADP, adenosine 5′-diphosphate; PEP, phosphoenolpyruvate; F6P, fructose 6-phosphate; G6P, glucose 6-phosphate.

A widely targeted metabolomics analysis was performed to assess the changes in metabolites in pigeonpea roots subjected to −Pi treatment and +Pi treatment. From this, 609 metabolites were identified and classified into 12 chemical compound categories (Supplementary Data Table S10). Among these metabolites, 173 were found to be differentially accumulated metabolites (DAMs), 77 of which were upregulated and 96 were downregulated (Supplementary Data Fig. S3a). A total of 37 P-containing metabolites were significantly reduced by Pi deficiency in pigeonpea roots, including 22 lipids and their derivatives, 8 nucleotides and their derivatives, and 7 sugars and their derivatives (Fig. 2a). More importantly, among these P-containing metabolites, the accumulation of phosphoethanolamine (PEth), adenosine 3′,5′-cyclic phosphate (cAMP), and phosphocholine (PCho) decreased the most during Pi starvation (Fig. 2a).

Subsequently, a lipidomic analysis was performed to assess the changes in lipids in pigeonpea roots following −Pi and +Pi treatments. As a result, a total of 924 lipids were identified, with 45 upregulated and 72 downregulated (Supplementary Data Fig. S3b; Supplementary Data Table S11). Among the downregulated lipids, 68% (49 of 72) were phospholipids, including 12 phosphatidylethanolamines (PEs), 8 phosphatidylglycerols (PGs), 4 phosphatidylcholines (PCs), 2 phosphatidic acids (PAs), two phosphatidylserines (PSs), 12 lysophosphatidylcholines (LPCs), 3 lysophosphatidylethanolamines (LPEs), 2 lysophosphatidylglycerols (LPGs), 2 lysophosphatidic acids (LPAs), and 2 lysophosphatidylinositols (LPIs) (Fig. 2b).

In addition, the concentration variation of adenosine 5′-triphosphate (ATP), adenosine 5′-diphosphate (ADP), phosphoenolpyruvate (PEP), fructose 6-phosphate (F6P), and glucose 6-phosphate (G6P) under −Pi and +Pi conditions were determined by liquid chromatography–tandem mass spectrometry (LC–MS/MS). The results showed that −Pi treatment resulted in 84–94% reduction in the concentrations of these five P-containing metabolites (Fig. 2c).

### Identification of genes involved in scavenging and recycling of organic P pools in pigeonpea

**Figure 3:**
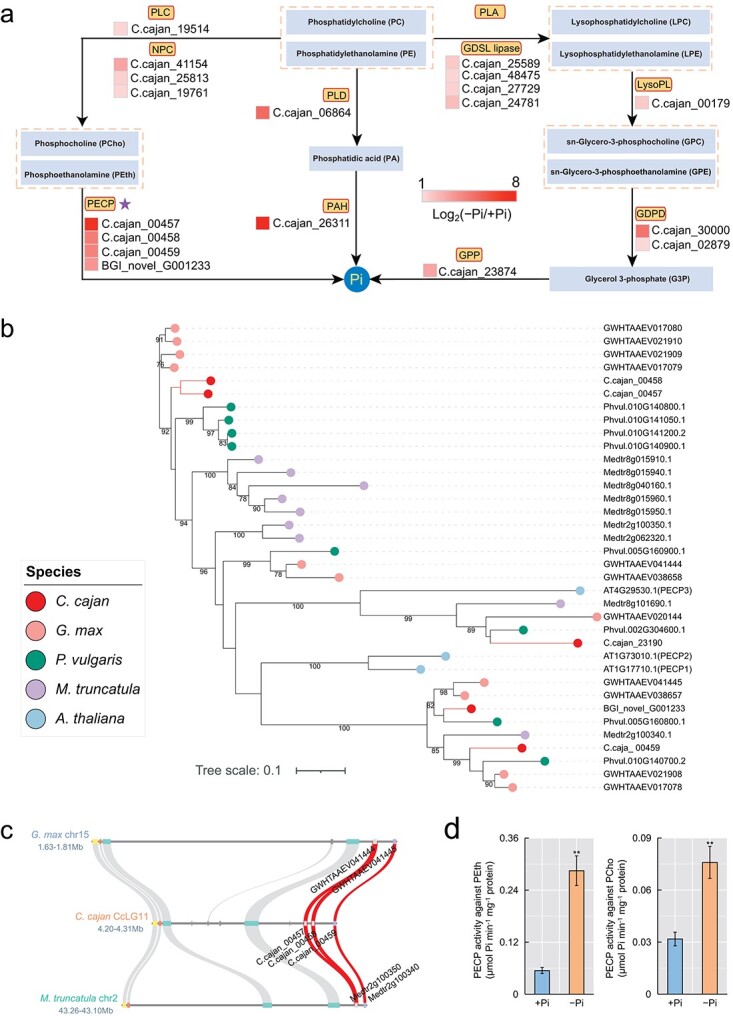
Overview of genes related to phospholipid degradation. **a** Changes in the expression of genes encoding enzymes involved in the phospholipid degradation pathway in the roots of pigeonpea seedlings grown under +Pi versus −Pi conditions. Log_2_(−Pi/+Pi) of upregulated genes is shown as a heat map. PLC, phospholipase C; NPC, non-specific phospholipase C; PLD, phospholipase D; PAH, phosphatidate phosphohydrolase; PECP, phosphoethanolamine/phosphocholine phosphatase; LysoPL, lysophospholipase; GDPD, glycerophosphodiester phosphodiesterase; GPP, glycerol-3-phosphatase. **b** Phylogenetic tree analysis of PECPs in pigeonpea (*C. cajan*), soybean (*G. max*), barrel medic (*M. truncatula*), common bean (*P. vulgaris*), and *Arabidopsis* (*A. thaliana*). Different colored circles represent different species. **c** Microcollinearity of tandemly duplicated *PECP*s in pigeonpea compared with soybean and barrel medic. *PECP*s are marked with their corresponding gene IDs. **d** PECP activity in the roots of pigeonpea. Data are means ± standard error of three biological replicates. Asterisks indicate significant differences between −Pi and +Pi treatments according to Student’s *t*-test: ^**^.001 ≤ *P* < .01.

The accumulation of phospholipids was significantly decreased in Pi-deficient pigeonpea roots. Hence, genome-wide identification of genes involved in phospholipid degradation was performed to investigate their response to Pi starvation. In our transcriptome analysis, genes involved in three phospholipid degradation pathways were significantly upregulated under low-Pi conditions, including one *phospholipase C* (*PLC*), three *non-specific phospholipase C* (*NPC*s), four *phosphoethanolamine*/*phosphocholine phosphatase*s (*PECP*s), one *phospholipase D* (*PLD*), one *phosphatidate phosphohydrolase* (*PAH*), four *GDSL lipase*s, one *lysophospholipase* (*LysoPL*), two *glycerophosphodiester phosphodiesterases* (*GDPD*s), and one *glycerol-3-phosphatase* (*GPP*) (Fig. 3a).

Most notably, KOG enrichment analysis of upregulated TDGs revealed that *haloacid dehalogenase-like hydrolase* (*HAD*) was significantly enriched (*P*_adj_ < .05; Fig. 1c), and the genes in this category encoded *PECP*s. Furthermore, we identified five *PECP*s in the pigeonpea genome (Table 1; Supplementary Data Table S12). Phylogenetic tree analysis and multicollinearity comparison of *PECP*s revealed that there was one more copy of *PECP*s on CcLG11 of pigeonpea, which occurred by tandem duplication ~16.13 million years ago (MYA) (Table 2; Fig. 3b and c). More importantly, four *PECP*s (including three tandem-duplicated *PECP*s) were upregulated in roots of P-deficient pigeonpea, and the fold change ranged from 10 to 160 (Fig. 3a; Supplementary Data Table S3). In addition, consistent with the upregulation of *PECP* genes under low-Pi conditions, −Pi treatment resulted in a significant increase in PECP activity in Pi-deficient pigeonpea roots (Fig. 3d).

**Table 1:** Number and classification of studied genes in pigeonpea

Gene name	Total number	DEG number	Up_DEG number	TDG number	Up_TDG number
*PECP*	5	4	4	3	3
*PAP*	30	14	14	4	4
*FLA*	35	15	15	12	8
*PHT1*	12	8	8	4	3
*GAD*	8	4	4	4	3

**Table 2:** Synonymous substitutions per synonymous site (*K*_s_) and estimated divergence time of paralogous gene pairs in pigeonpea

Gene name	Paralog gene pairs	Type	*K*_s_	Divergence time (MYA)
*PECP*	C.cajan_00457 vs C.cajan_00458	Tandem	0.1968	16.13
*PECP*	C.cajan_00458 vs C.cajan_00459	Tandem	1.6759	137.37
*PAP*	C.cajan_06122 vs C.cajan_06164	Dispersed	0.0523	4.29
*PAP*	C.cajan_23091 vs C.cajan_23092	Tandem	1.9889	163.02
*PAP*	C.cajan_10069 vs C.cajan_10070	Tandem	2.6319	215.73
*FLA*	C.cajan_21485 vs C.cajan_21486	Tandem	0.319	26.15
*FLA*	C.cajan_21503 vs C.cajan_21504	Tandem	0.2542	20.84
*FLA*	C.cajan_21504 vs C.cajan_21505	Tandem	0.2648	21.70
*FLA*	C.cajan_21505 vs C.cajan_21506	Tandem	1.2995	106.52
*FLA*	C.cajan_21506 vs C.cajan_21507	Tandem	1.1626	95.30
*FLA*	C.cajan_32261 vs C.cajan_32262	Tandem	0.0999	8.19
*PHT1*	C.cajan_41061 vs C.cajan_26900	Dispersed	0.03	2.46
*PHT1*	C.cajan_34792 vs C.cajan_28378	Dispersed	0.0649	5.32
*PHT1*	C.cajan_34791 vs C.cajan_34792	Tandem	0.3635	29.8
*PHT1*	C.cajan_22562 vs C.cajan_22563	Tandem	1.4241	116.73
*GAD*	C.cajan_23036 vs C.cajan_23037	Tandem	0.0992	8.13
*GAD*	C.cajan_19621 vs C.cajan_19625	Tandem	0.0073	0.60

Moreover, genes involved in RNA, DNA, and ATP degradation were identified; as a result, three *S-like ribonuclease* (*RNS*) genes, one *nuclease DPD1* gene, and one *apyrase* (*APY*) gene were upregulated in Pi-deficient pigeonpea roots (Supplementary Data Fig. S4a). P-containing nucleotides and their derivatives resulting from the degradation of RNA, DNA, and ATP can be further dephosphorylated by purple acid phosphatases (PAPs) [17]. There are 30 *PAP* genes in the pigeonpea genome (Supplementary Data Table S13). Phylogenetic tree analysis of PAPs showed that the subclass of PAPs was conserved in pigeonpea, barrel medic (*Medicago truncatula*) and common bean (*Phaseolus vulgaris*), and that two tandem-duplicated *PAP*s in the pigeonpea genome occurred anciently (Table 2; Supplementary Data Fig. S4b). Most notably, 46.67% (14 out of 30) *PAP* genes in the roots of pigeonpea were upregulated under low-Pi stress (Supplementary Data Fig. S4c).

### Identification of *fasciclin-like arabinogalactan protein* and *expansin* gene families in pigeonpea

**Figure 4:**
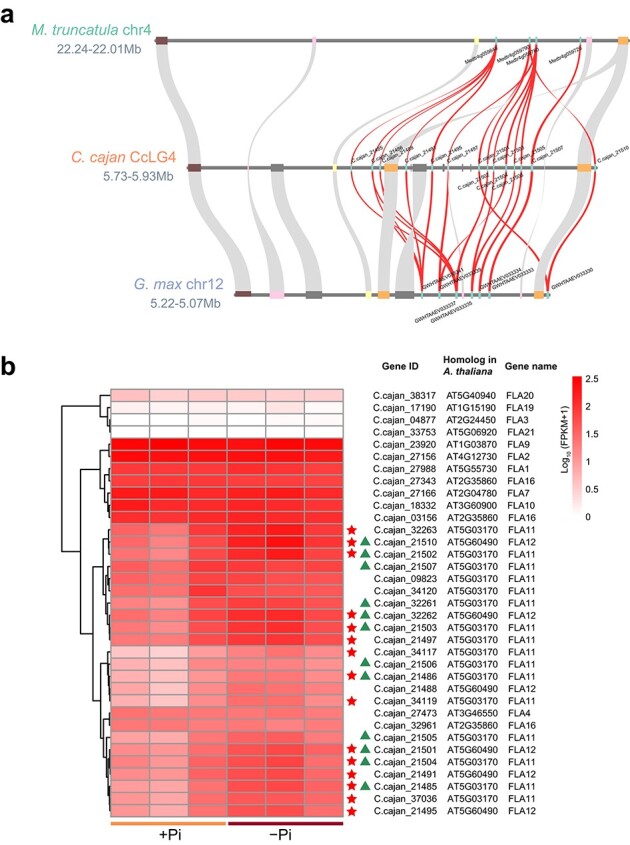
Microcollinearity and gene expression of *fasciclin-like arabinogalactan protein* genes (*FLA*s). **a** Microcollinearity of tandemly duplicated *FLA*s in pigeonpea (*C. cajan*) compared with soybean (*G. max*) and barrel medic (*M. truncatula*). *FLA*s are marked with their corresponding gene IDs. **b** Gene expression heat map of *FLA*s in pigeonpea roots under +Pi and −Pi conditions. Upregulated DEGs are marked with red stars and TDGs are marked with triangles.

In this study, *arabinogalactan proteins* (*AGPs*) were significantly enriched in both TDGs and upregulated TDGs following KOG enrichment analysis (*P*_adj_ < 0.05; Fig. 1c). Fasciclin-like arabinogalactan proteins (FLAs) are a subclass of AGPs, and genes enriched in terms of *AGPs* were *FLA*s (Fig. 1c; Supplementary Data Table S14). Hence, genome-wide identification of *FLA*s was performed, and a total of 36 *FLA*s were identified in pigeonpea, with similar numbers in barrel medic (32) and common bean (39) (Supplementary Data Table S14). Phylogenetic tree analysis and multicollinearity comparison of *FLA*s showed that there were more copies of *FLA*s on CcLG4 of pigeonpea (three pairs of *FLA*s), which were expanded by recent tandem duplication (divergence time ~20.84–26.15 MYA; Fig. 4a; Supplementary Data Fig. S5; Table 2). In addition, there was another pair of *FLA*s (C.cajan_32261 vs C.cajan_32262) expanded by recent tandem duplication (~8.19 MYA; Table 2). More importantly, 15 *FLA*s were upregulated in response to Pi starvation, 8 and 5 of which were TDGs and recent expanded TDGs, respectively (Fig. 4b; Table 2). In addition, a total of 39 *expansin* genes were identified in pigeonpea, with similar numbers in common bean (38) and *Arabidopsis* (35) (Supplementary Data Table S15). There were three upregulated and two downregulated *expansin* genes under low-Pi conditions (Supplementary Data Fig. S6).

### Comprehensive analysis of *phosphate transporter 1* gene family in pigeonpea

**Figure 5:**
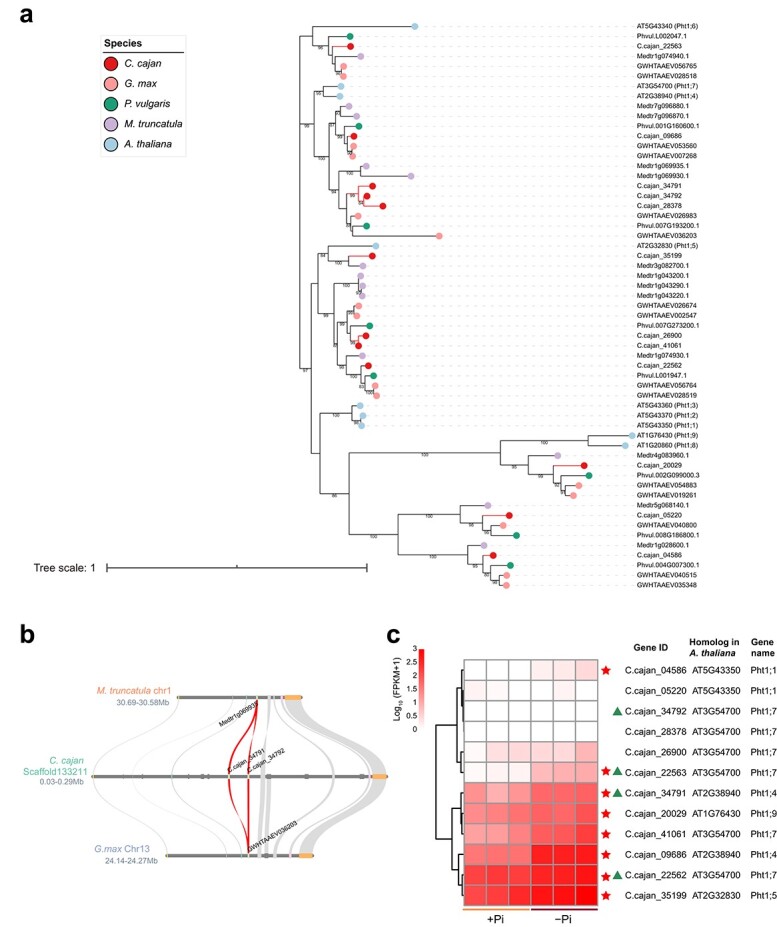
Gene collinearity, evolution, and expression of *PHT1*s. **a** Phylogenetic tree analysis of *PHT1*s in pigeonpea (*C. cajan*), soybean (*G. max*), common bean (*P. vulgaris*), barrel medic (*M. truncatula*), and *Arabidopsis* (*A. thaliana*). Different colored circles represent different species. **b** Microcollinearity of tandemly duplicated *PHT1*s in pigeonpea compared with soybean and barrel medic. *PHT1*s are marked with gene IDs. **c** Gene expression heat map of *PHT1*s in pigeonpea roots under +Pi and −Pi conditions. Upregulated DEGs are marked with red stars and TDGs are marked with triangles.

*Phosphate transporter 1* (*PHT1*) genes were significantly enriched in upregulated DEGs, TDGs, and upregulated TDGs by GO and KOG enrichment analysis (Fig. 1a and c; Supplementary Data Tables S4 and S7–S9). There were 12, 15, 13, and 8 *PHT1*s in pigeonpea, soybean, barrel medic, and common bean, respectively (Supplementary Data Table S16). More importantly, there were more *PHT1;7*s in pigeonpea (six *PHT1;7*s) than in the other four studied plant species (Supplementary Data Table S16). A total of three paralogous pairs of *PHT1*s (one pair of tandem-duplicated and two pairs of dispersed-duplicated genes) had expanded recently (divergence time ~2.46–29.8 MYA; Table 2). Phylogenetic tree analysis and multicollinearity comparison of *PHT1*s showed that the recent expansion of the *PHT1* gene family was a special event in pigeonpea (Fig. 5a and b). Furthermore, 8 out of 12 (66.67%) *PHT1*s were upregulated in −Pi conditions compared with +Pi conditions, three of which were TDGs (Fig. 5c).

### Changes in metabolism and secretion of carboxylates in Pi-deficient pigeonpea roots

**Figure 6:**
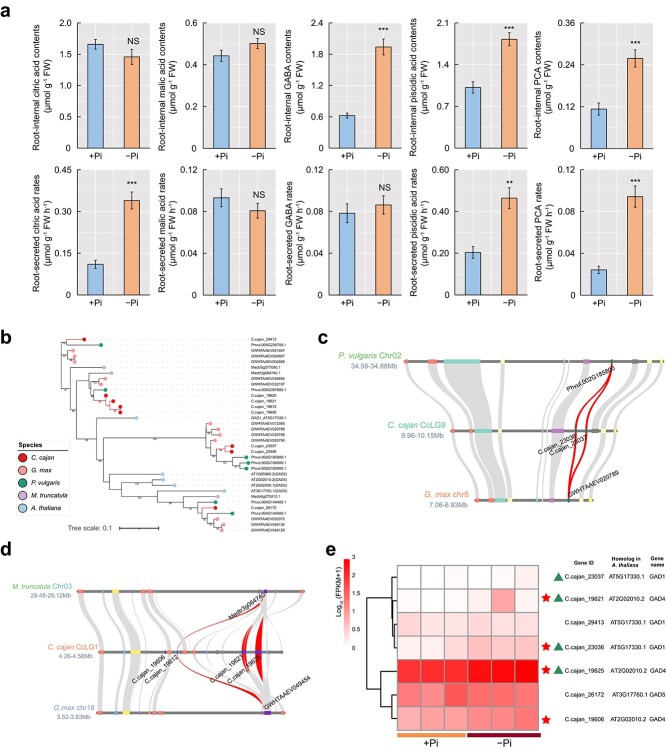
PSR of carboxylate synthesis and secretion in pigeonpea roots and gene family analysis of *GAD*s. **a** Endogenous and secreted contents of five carboxylates in roots of pigeonpea seedlings grown under +Pi or −Pi conditions. Data are means ± standard error of five biological replicates. Asterisks indicate significant differences between −Pi treatment and +Pi treatment according to Student’s *t*-test: ^**^.001 ≤ *P* < .01; ^***^P < .001; NS, not significant. **b** Phylogenetic tree analysis of GADs in pigeonpea (*C. cajan*), soybean (*G. max*), common bean (*P. vulgaris*), barrel medic (*M. truncatula*), and *Arabidopsis* (*A. thaliana*). Different colored circles represent different species. **c** Microcollinearity of tandemly duplicated *GAD*s in pigeonpea compared with common bean and soybean; the corresponding collinearity regions were absent in barrel medic. *GAD*s are marked with their corresponding gene IDs. **d** Microcollinearity of tandemly duplicated *GADs* in pigeonpea compared with barrel medic and soybean; the corresponding collinearity regions were absent in common bean. *GAD*s are marked with their corresponding gene IDs. **e** Gene expression heat map of *GADs* in pigeonpea roots under +Pi and −Pi conditions. Upregulated DEGs are marked with red stars and TDGs are marked with triangles.

It has been reported that pigeonpea is extremely efficient in utilizing insoluble P in soils [18]. Root-secreted carboxylates are thought to play a crucial role in the hydrolysis of insoluble P [19]. In this study, five carboxylates were analyzed for their amount of endogenous and secreted contents in the roots of pigeonpea seedlings grown under +Pi or −Pi conditions, including citric acid, malic acid, piscidic acid, γ-aminobutyric acid (GABA), and protocatechuic acid (PCA). The results showed that root-internal citric acid contents and root-internal malic acid contents were not significantly different between −Pi and + Pi treatments (Fig. 6a). In accordance with this result, *citrate synthase* (*CSY*) and *malate dehydrogenase* (*MDH*), involved in the synthesis of citric acid and malic acid, respectively, were not influenced by Pi starvation (Supplementary Data Fig. S7). In contrast, −Pi treatment significantly increased the accumulation of root-internal piscidic acid, GABA, and PCA (Fig. 6a). In addition, −Pi treatment also resulted in remarkable increases in root-secreted citric acid, piscidic acid, and PCA (Fig. 6a). Multidrug and toxic compound extrusion transporter (MATE) and aluminum-activated malate transporter (ALMT) are two transporter families involved in the efflux of root carboxylates in plants [19], so we analyzed the expression of *MATE* and *ALMT* family genes in the transcriptome profiles. As a result, we identified four *MATEs* and one *ALMT* upregulated by Pi starvation (Supplementary Data Fig. S8).

In this study, *glutamate decarboxylase* (*GAD*) was significantly enriched in both TDGs and upregulated TDGs by KOG enrichment analysis (*P*_adj_ < .05; Fig. 1c). The main function of GAD is to synthesize GABA. There were more *GAD*s (eight) in pigeonpea than in barrel medic, common bean, and *Arabidopsis* (Supplementary Data Table S17). Phylogenetic tree analysis and multicollinearity comparison of *GAD*s showed that there were two pairs of tandem-duplicated *GAD*s with divergence time ~0.60 and 8.13 MYA in the pigeonpea genome (Fig. 6b–d; Table 2). More importantly, four *GAD*s were upregulated under low-Pi conditions, three of which were TDGs (Fig. 6e).

## Discussion

Pigeonpea is a legume crop with excellent adaptability to nutrient-poor soils, and in particular it is extremely tolerant of Pi deficiency [18]. The release of the pigeonpea genome sequence provides a foundation to investigate the molecular mechanisms of low-Pi stress tolerance in this species [14]. Although there has been no recent whole-genome duplication event, the 605-Mb genome assembly of pigeonpea contains 48 680 predicted protein coding genes [14], which reveals that there are other events leading to gene duplications in the pigeonpea genome, such as tandem duplication and segmental duplication. In this study, several gene families expanded through tandem duplication were upregulated in pigeonpea roots under low-Pi conditions (Fig. 1c; Supplementary Data Table S6). There may be a crucial role for the TDGs in the development of low-Pi tolerance traits in pigeonpea.

*FLA*s are a class of cell wall structural glycoproteins that belong to the hydroxyproline-rich glycoprotein (HRGP) superfamily [20]. It has been documented that *FLA*s are involved in mediating plant growth, development, and response to abiotic stresses [20]. *FLA*s have been reported to play an important role in root development [21]. For example, *AtFLA18* regulates root elongation in *Arabidopsis*, and mutations of *AtFLA18* result in shorter lateral roots [21]. *BcFLA1* from *Brassica carinata* is reported to be involved in the regulation of root hair development under low-Pi conditions [22]. In our study, six paralogous pairs of *FLA*s occurred by tandem duplication, four of which had expanded recently (Table 2). More importantly, 15 *FLA*s were upregulated in response to Pi starvation, 5 of which were recently expanded TDGs (Fig 4b; Table 2). The expansion of *FLA*s in the pigeonpea genome may have an important contribution to the remodeling of root morphology under low-Pi stress. In agreement with this hypothesis, low-Pi treatment for 14 days promoted root growth in pigeonpea, resulting in a significant increase in the total root length, root surface area, and root-to-shoot ratio of pigeonpea (Supplementary Data Fig. S1).

Phospholipids generally account for about one-third of the organically bound P in plant tissues [23]. The conversion of phospholipids to non-P-containing lipids (e.g. galactolipids and sulfolipids) as well as the release of Pi are major responses that have been shown to occur under conditions of Pi deficiency in many plant species [24]. Similarly, in this study, metabolomic and lipidomic analyses showed that low-Pi treatment resulted in decreased accumulation of phospholipids and increased accumulation of galactolipids and sulfolipids [e.g. monogalactosyldiacylglycerol (MGDG), digalactosyldiacylglycerol (DGDG), and sulfoquinovosyl diacylglycerol (SQDG)] in pigeonpea roots (Fig. 2). Genome-wide identification of genes involved in phospholipid degradation found that three *PECP*s (*AtPECP1*-homologous genes) in pigeonpea occurred by tandem duplication, two of which had expanded recently (Fig. 3b and c; Table 2). It has been demonstrated that AtPECP1 can hydrolyze PEth and PCho in *Arabidopsis* when subjected to Pi starvation [25]. Our results suggest that these three tandemly duplicated *PECP*s of pigeonpea may perform a similar function to that of *AtPECP1*, as reflected by the upregulation of three tandem-duplicated *PECP*s under low-Pi conditions, as well as the increase in PECP activity, and the reduction of PEth and PCho contents in Pi-deficient pigeonpea roots (Fig. 3). These results suggest that pigeonpea *PECP*s may cooperate with other genes involved in phospholipid degradation to enhance Pi recycling from phospholipids under low-Pi conditions (Fig. 3).

The soil contains large amounts of insoluble P (e.g. Fe-P, Al-P), which cannot be absorbed directly by the plant roots. It has been demonstrated that piscidic acid secreted by the roots of pigeonpea can efficiently degrade Fe-P and release Pi for root uptake [18]. The present study also verified this conclusion that root-secreted piscidic acids were increased in P-deficient pigeonpea (Fig. 6a). In addition to piscidic acids, we also found that low-Pi treatment promoted more citric acid and PCA secretion from the roots of pigeonpea (Fig. 6a). MATE transporters are responsible for the secretion of citric acid from roots in plants [26]. Furthermore, the MATE transporter was also reported to be the efflux transporter of PCA [26]. For example, two members of the MATE transporter family in rice, namely OsPEZ1 and OsPEZ2, have been demonstrated to be primarily responsible for PCA efflux from roots [27]. In the present study, four *MATEs* were upregulated in P-deficient pigeonpea roots (Supplementary Data Fig. S8a), and they may be involved in the secretion of citric acid and PCA from the roots of pigeonpea. GABA has been shown to be a signaling molecule involved in regulating plant adaptation to various environmental stresses [28]. A recent study has shown that *Liriodendron* regulates the expression of *MATE* genes and promotes citric acid secretion by increasing the synthesis of GABA, when subjected to toxic aluminum stress [29]. In the pigeonpea genome, four *GAD* genes (encoding enzymes responsible for GABA synthesis) were expanded by tandem duplication recently (Fig. 6). Low-Pi treatment resulted in an increased expression of *GAD*s in pigeonpea roots, accompanied by an increase in endogenous GABA accumulation (Fig. 6). Based on these findings, we speculate that the GADs in pigeonpea may regulate the expression of *MATE* genes by synthesizing GABA under low-Pi conditions, thus regulating the secretion of citric acid and PCA in pigeonpea roots.

As well as insoluble P, organic P cannot be absorbed directly by plant roots. It has been estimated that organic P accounts for 50% of the total P in the upper layers of soils [30]. Plant PAPs can be secreted from the roots into the rhizosphere, and play an important role in degrading soil organic P and releasing Pi [31]. In the present study, 14 out of the 30 *PAP* genes (46.67%) were upregulated in P-deficient pigeonpea roots, and were accompanied by increases in root-internal APase and root-associated APase activities (Supplementary Data Figs S1 and S4). These results suggest that pigeonpea may enhance organic P utilization by inducing the expression of *PAP*s when subjected to low-Pi stress. In addition, PHT1 family members are the main Pi transporters responsible for Pi uptake from the soil in plant roots [32]. In the pigeonpea genome, five *PHT1*s expanded recently through tandem duplication or dispersed duplication (Table 2; Fig. 5). At the transcriptional level, >66.66% (8 out of 12) *PHT1*s were upregulated in −Pi conditions compared with +Pi conditions, including the recent expanded tandem- and dispersed-duplicated *PHT1*s (Fig. 5c). Under low-Pi conditions, the increased expression of *PHT1s* in pigeonpea roots facilitated the uptake of soil Pi, which included Pi release from insoluble P and organic P, hydrolyzed by root-secreted carboxylates and PAPs.

### Conclusions

In this study, integration of genome, transcriptome, and metabolite profiling analyses was performed to investigate gene evolution and modulations in gene expression and metabolite accumulation in response to Pi deficiency in pigeonpea. Four gene families that were upregulated in P-deficient roots were expanded by tandem duplication in the pigeonpea genome; these include *PECP*s , *PHT1*s, *FLA*s, and *GAD*s. These four gene families may be involved in regulating PSR of pigeonpea, such as phospholipid degradation, Pi uptake, root morphological remodeling, and root carboxylate secretion, thus helping pigeonpea to increase PAE and PUE under low-Pi conditions. Overall, our present study contributes to the understanding of the diverse responses and adaptations of pigeonpea to low-Pi stress.

## Materials and methods

### Plant growth and treatment

The seeds of pigeonpea (*C. cajan*) genotype CF052777 were provided by the Tropical Crops Genetic Resources Institute (TCGRI), Chinese Academy of Tropical Agricultural Sciences (CATAS), Hainan, China. Pigeonpea seeds were soaked in distilled water for 12 hours, followed by germination in paper rolls moistened with half-strength modified Magnavaca’s nutrient solution [33]. After 7 days of germination, seedlings were transplanted to a hydroponic box filled with modified Magnavaca’s nutrient solution and precultured for 10 days. Subsequently, uniform seedlings were transferred to new Magnavaca’s nutrient solution (pH 5.8) either with (+Pi, 0.3 mM KH_2_PO_4_) or without P (−Pi, 0 mM KH_2_PO_4_). During the experiment, the treatment solution was renewed every 3 days. After 14 days of treatments, shoots and roots were harvested separately for the determination of FW, DW, P content, and other analyses. Following root image acquisition from a scanner (Epson, Japan), total root length and root surface area were evaluated using image analysis software (WinRhizo Pro, Regent Instruments, Quebec, Canada). In all experiments, unless otherwise stated, there were three biological replicates, and each biological replicate contained 10 seedlings.

### Determination of total P content, APase activity, and PECP activity

Pigeonpea samples were oven-dried at 65°C, then the biomass was weighed. The dried samples were then ground into powder. Subsequently, ~0.05 g of powdered sample was burned to ash in a muffle furnace at 600°C. The ash samples were completely dissolved in 100 mM HCl. Finally, the supernatant was collected and used to determine the total P content, as described previously [34].

Measurement of root-internal APase activity was performed according to a previously published protocol with some modifications [35]. Briefly, liquid nitrogen was used to grind fresh samples into a fine powder. Then, the powdered samples (~0.1 g) were ground and homogenized in 50 mM Na-acetate buffer (pH 5.6) containing 1 mM EDTA, 1 mM phenylmethylsulfonyl fluoride, and 5 mM thiourea, on ice. The homogenates were centrifuged at 14 000 g for 20 min at 4°C, followed by collection of the supernatants for further analysis. An aliquot of 0.2 mL of supernatants was mixed with 0.6 mL of 50 mM Na-acetate buffer (pH 5.6) that contained 2 mM *para*-nitrophenyl-phosphate (*p*NPP) as the substrate. The mixture was then incubated for 15 minutes at 37°C. In order to terminate the reaction, 0.8 mL of 0.5 M NaOH was added. To quantify the amount of *para*-nitrophenol (*p*NP) released from *p*NPP, the absorbance of the reaction mixture was measured spectrophotometrically at 405 nm. The Coomassie Brilliant Blue method [36] was used to determine soluble protein concentration. Enzyme activity was given as units (U) per milligram of protein. One unit was defined as the release of 1 μmol *p*NP per minute. Measurement of root-associated APase activity was performed as described previously [37]. The activity of PECP was measured according to the published method [38].

### Transcriptome analysis

Three biological replicate samples of pigeonpea roots for +Pi and −Pi treatments were harvested for RNA sequencing using a DNBSEQ-T7 sequencer (MGI-Tech, China). Raw sequencing reads were filtered as low-quality, N- and adapter-containing reads using the filter module included in SOAPnuke (version 2.16). On the basis of the pigeonpea genome [14], clean reads were mapped onto genome sequences using HISAT2 (version 2.0.4) with sensitive parameters. Furthermore, StringTie (version 1.3.3b) was employed to reconstruct transcripts. CPC (Coding Potential Calculator) [39] was adopted to predict the protein coding capacity of novel genes, and genes with protein-coding capacity were retained for subsequent analysis. To get gene expression profiles, bowtie2 (version 2.2.5) was employed to map clean reads to the genes and transcript dataset, and RSEM (version 1.3.3) was adopted to calculate gene expression. In addition, differential gene expression analysis was carried out using DESeq2 (version 1.32.0) on the R platform (version 4.0.2), and genes with expression fold change ≥2 or ≤0.5 and adjusted *P*-value ≤.05 were considered as DEGs. Further KEGG pathway and GO enrichment analyses were performed using the *phyper* and *p.adjust* functions on the R platform (version 4.0.2). KOG enrichment analysis of upregulated TDGs and upregulated TDGs was performed using the *fisher.test* and *p.adjust* functions on the R platform (version 4.0.2). An adjusted *P*-value ≤.05 was regarded as significantly enriched.

### Identification and analysis of PSR genes

In order to identify PSR genes in the pigeonpea genome and other legumes, protein sequences of PSR genes (including *PHT1*s, *PAP*s, *FLA*s, *expansin*s, *CSY*s, *MDH*s, *MATE*s, *ALMT*s, and genes associated with degradation pathways of phospholipid, RNA, DNA, and ATP) in *A. thaliana* (The Arabidopsis Information Resource; www.arabidopsis.org) were used as seed sequences. Protein sequences from pigeonpea [14], soybean [40], barrel medic [41], and common bean [42] were aligned to the seed sequences using BLASTP (version 2.9.0) with *e*-value set to 1*e*^−10^. Genes with alignment identity ≥35% and coverage ≥35% were considered as candidate genes. To investigate the multicollinearity between pigeonpea and other studied plant species, MCscan [https://github.com/tanghaibao/jcvi/wiki/MCscan (Python version)] integrated in JCVI utility libraries was carried out to construct multi-synteny blocks, and the interested region was visualized. Protein sequences of identified genes from pigeonpea and three other studied plant species as well as *Arabidopsis* were performed with multiple sequence alignment using Muscle (version 3.8.31), and a maximum-likelihood phylogenetic tree of each gene family was reconstructed by FastTree [43] (version 2.1.10) with a JTT model. Finally, iTOL (https://itol.embl.de/) was employed to visualize the phylogenetic tree.

### Calculation of synonymous substitutions per synonymous site (*K*_s_) and estimation of divergence time

To investigate the TDGs in pigeonpea, a method described in our previous report was adopted [44]. In brief, protein sequences from pigeonpea were self-aligned by BLASTP (version 2.9.0) and then MCScanX [45] was carried out to analyze the BLAST results. On the basis of results from MCScanX, TDGs were identified with code for gene type appearing as ‘3’, and dispersed and proximal duplicated genes were identified with code for gene type appearing as ‘1’ and ‘2’, respectively. Furthermore, the *K*_s_ value of paralogous gene pairs from TDGs and genes clustered in the phylogenetic tree was calculated using PAML (version 4.9e) using the Nei–Gojobori method [46]. The estimated divergence time of each paralogous gene pair was calculated by the formula *T* = *K*_s_/2*r*, and a neutral substitution rate of 6.1 × 10^−9^*K*_s_ year^−1^ was adopted [47] in the current study.

### Metabolomic analysis, lipidomic analysis, and targeted metabolite detection

After sample treatment and collection, a widely targeted metabolomics analysis was conducted at MetWare Biotechnology Co. Ltd (Wuhan, China) on the basis of a previously described method [12]. In brief, samples were ground into powder and then 100 mg powder was extracted in 1.0 mL of 70% aqueous methanol overnight at 4°C. Next, the extract was absorbed and filtered for further analysis using a UPLC–ESI–MS/MS system. This system was a combination of UPLC (Shim-pack UFLC, Shimadzu CBM30A system) and MS (Applied Biosystems 4500 Q TRAP). The conditions for the UPLC and MS were as described previously [12]. The MetWare database (MWDB) was used to identify the metabolites. The abundance of metabolites was calculated based on their peak areas. Metabolites with change in relative abundance >2-fold between −Pi and +Pi treatments, as well as variable importance in project (VIP) >1.0, were defined as DAMs. Lipidomic analysis was conducted at MetWare Biotechnology Co. Ltd (Wuhan, China) according to previously described methods [48].

Targeted determination of ATP, ADP, phosphoenolpyruvate (PEP), fructose 6-phosphate (F6P), and glucose 6-phosphate (G6P) using an LC–MS/MS system was performed at Shanghai Applied Protein Technology Co. Ltd (Shanghai, China) based on published methods [49]. Each treatment in this experiment had five biological replicates.

### Detection of endogenous and secreted carboxylates

Pigeonpea seedlings were grown in nutrient solution with (+Pi, 0.3 mM KH_2_PO_4_) or without P (−Pi, 0 mM KH_2_PO_4_) for 14 days as described above. To collect secreted carboxylates (citric acid, malic acid, piscidic acid, GABA, and PCA), seedlings were transferred to centrifuge tubes containing 45 mL of 0.5 mM CaCl_2_ collecting solution for 12 hours. Using a freeze-drying vacuum system (Labconco, Kansas, MO, USA), the collected solution was concentrated into dry powder before being redissolved in Millipore water for measurement. For the extraction of internal citric acid, malic acid, piscidic acid, and PCA, 0.2-g root samples were ground with 1.5 mL of 0.25 M HCl, and the homogenate was then heated to 80°C for 20 minutes with intermittent shaking. After centrifugation at 14000 g for 20 minutes, the supernatants were collected for further analysis. All samples were passed through a filter (0.45 μm). The contents of citric acid, malic acid, piscidic acid, and PCA were analyzed with an HPLC 1260 Infinity LC (Agilent, USA), as previously described [16, 50]. Extraction of endogenous GABA and determination of GABA content were performed according to a previously published method [29]. Each treatment in this experiment had five biological replicates.

## Acknowledgements

The research was financially supported by the Young Elite Scientists Sponsorship Program by CAST (2019QNRC001), the Agricultural Research Outstanding Talents and Innovation Team of MARA (13210268), the China Agriculture Research System of MOF and MARA (CARS-34; CARS-22), the Natural Science Foundation of Hainan Province (320RC729), the Program of Hainan Association for Science and Technology Plans to Youth R & D Innovation (QCXM201901), the Central Public-interest Scientific Institution Basal Research Fund for CATAS (1630032020003; 1630032022023), and the Science, Technology and Innovation Commission of Shenzhen Municipality (JCYJ20180507183534578).

## Author contributions

P.L. and G.L. conceived the research. C.L. and Y.T. performed bioinformatics analysis. J.L., Y.W., and X.Z. performed the physiological experiments. L.L., R.D., and X.D. provided experimental materials and resources. S.Z. provided technical support and suggestions on manuscript revision. P.L., G.L., C.L., and Y.T. discussed the data and wrote the paper. All authors read and approved the final manuscript.

## Data availability

The transcriptome sequencing data are available in the Genome Sequence Archive (GSA) in the National Genomics Data Center, under Bioproject accession number PRJCA007387 and GSA accession number CRA005471.

## Conflict of interest

The authors declare that they have no conflict of interest.

## Supplementary data

Supplementary data is available at *Horticulture Research* online.

## Supplementary Material

Web_Material_uhac107
